# Modeling, Control, and Simulation of Battery Storage Photovoltaic-Wave Energy Hybrid Renewable Power Generation Systems for Island Electrification in Malaysia

**DOI:** 10.1155/2014/436376

**Published:** 2014-04-30

**Authors:** Nahidul Hoque Samrat, Norhafizan Bin Ahmad, Imtiaz Ahmed Choudhury, Zahari Bin Taha

**Affiliations:** ^1^Centre for Product Design and Manufacturing (CPDM), Department of Mechanical Engineering, Faculty of Engineering, University of Malaya, 50603 Kuala Lumpur, Malaysia; ^2^Innovative Manufacturing, Mechatronics and Sports Laboratory (iMAMS), Faculty of Manufacturing Engineering, Universiti Malaysia Pahang, Pekan, 26600 Pahang, Malaysia

## Abstract

Today, the whole world faces a great challenge to overcome the environmental problems related to global energy production. Most of the islands throughout the world depend on fossil fuel importation with respect to energy production. Recent development and research on green energy sources can assure sustainable power supply for the islands. But unpredictable nature and high dependency on weather conditions are the main limitations of renewable energy sources. To overcome this drawback, different renewable sources and converters need to be integrated with each other. This paper proposes a standalone hybrid photovoltaic- (PV-) wave energy conversion system with energy storage. In the proposed hybrid system, control of the bidirectional buck-boost DC-DC converter (BBDC) is used to maintain the constant dc-link voltage. It also accumulates the excess hybrid power in the battery bank and supplies this power to the system load during the shortage of hybrid power. A three-phase complex vector control scheme voltage source inverter (VSI) is used to control the load side voltage in terms of the frequency and voltage amplitude. Based on the simulation results obtained from Matlab/Simulink, it has been found that the overall hybrid framework is capable of working under the variable weather and load conditions.

## 1. Introduction

In developing countries like Malaysia, the development of islands is mostly related to the electric power availability, because there are many islands all over Malaysia where electric power grid is not available. Among these island communities electricity is supplied by traditional energy sources, but the fuel cost increases significantly with remoteness. Furthermore, the energy produced by the conventional sources raises the greenhouse gas emissions, which may be the key source of global warming. It is projected that, by 2020, Malaysia will release 285.73 million tons of CO_2_ which is an increase of 68.86% compared to the amount of CO_2_ emitted in the year 2000. In Malaysia, electricity generation alone contributes 43.40% of the total CO_2 _emission, which is the largest among all sectors [1]. Malaysia signed the previous Kyoto protocol on reduction of CO_2_ emission to the atmosphere. For this reason, the Malaysian government is very much concerned about environmental issue and the government wants the overall improvement of the CO_2_ emission. As a result, island electrification in Malaysia by renewable energy sources is the only way to overcome the challenge.

Among the renewable energy sources, solar energy is an environmentally friendly and the fastest growing green energy source. But the main drawback of the PV system is that the power produced by this system is highly dependent on climatic conditions. For example, a PV system could not able to produce any power at night and during cloudy periods. So the PV system intermittently produces power, which means that PV system may not totally satisfy the load demand at each instant. This problem can be solved by combining PV system with other renewable energy sources and/or energy storage systems (such wind, wave, fuel cell, battery bank, ultracapacitor bank, and hydrogen storage tank) in a suitable hybrid framework [2–7]. As an island surrounded by sea, wave energy can be considered one of the environmentally friendly hybrid power generating sources for island communities.

Wave energy is a renewable energy generated by the force of surface waves from the ocean. Although many wave energy conversion techniques have been patented and new patents are granted each month [8], there are only nine basic techniques on which these conversions are based. The nine basic techniques are cavity resonators or oscillating water column, pressure devices, heaving and pitching bodies, Salter's duck, surging wave energy converters, particle motion converters, Russell's rectifier, Cockerell's rafts, and wave focusing techniques [9–11]. In this study, an OWC wave energy converter device is preferred because OWC is generally considered one of the most promising wave energy conversion devices among the various wave energy converters [12]. However, assisting PV-wave hybrid system with battery banks makes economic sense when satisfying the transient's period or peak load demands. Battery based energy storage system is widely used in standalone system because of its mature technology, high efficiency, quick response, and low cost [13, 14]. Without battery bank, the PV-wave hybrid system must meet all load demands, thus increasing the cost and size of the hybrid system.

An extensive review based on the solar and other relevant areas has been reported in the literature to model hybrid renewable energy system. Among them, Onar et al. [6, 7] described detail dynamic model, mathematical modeling, and simulation of both solar/fuel cell/ultracapacitor and wind/fuel cell/ultracapacitor hybrid system. In [15], a wind, PV, and wave based large-scale hybrid system integration was analyzed and grid connection was discussed. Bhende et al. [13] investigated a standalone fuel cell based wind energy supply system. Power conditioning for hydrogen storage based wind energy system has been reported in [16]. In [2, 6, 7, 13, 15], the authors are silent about the wave energy based hybrid system design for island communities.

In this paper, detailed modeling, control, and simulation of a PV-wave hybrid renewable power generation system are developed for island communities. OWC wave energy device is used to generate the electrical power from the sea waves and PV model is used to generate power from solar radiation. A control algorithm is developed using a BBDC between the battery bank and dc-link, and a switch mode inverter is placed at the load side end. A simple passive L-C filter is placed after the inverter at load side end to eliminate the unwanted high frequency harmonics, which are generated by the load side VSI based on the inverter switching frequency.

The simulation model can be used not only for analyzing the battery storage based PV-wave hybrid system performance, but also for designing and sizing the system HRES to meet the consumer load demands for any available meteorological condition. The proposed standalone PV-wave hybrid system model in this paper has been modeled, designed, and simulated using Matlab, Simulink, and SimPowerSystems software packages. In addition, simulation results are presented to verify the effectiveness of the proposed system under variable weather conditions.

The sequential workflow hints of this paper are as follows. In Section 2 the complete modeling process of PV-wave hybrid system has been described with the necessary mathematical equations. And also Section 2 presents the control algorithm of dc-link voltage and load side VSI. In Section 3, according to the meteorological data, Perhentian Island is considered as a potential area for generating electric power from PV and wave energy sources. In Section 4 all the necessary simulation results and discussions are given to check the feasibility of the hybrid system. Finally, a conclusion has been drawn by combining all the important points of the study in Section 5.

## 2. System Description

**Figure 1:**
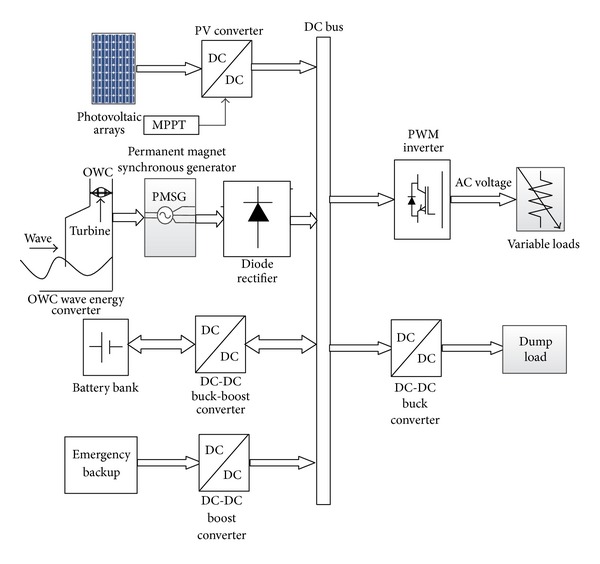
Block diagram of the proposed standalone PV-wave hybrid system.

In this section, the detailed simulation model of PV-wave hybrid renewable power generation system is briefly described.  Figure 1 shows the complete block diagram of the standalone PV-wave HRES. The developed hybrid system consists of five main parts: PV system, OWC system, battery bank, a BBDC with proportional integral (PI) control duty cycle, and a pulse width modulation (PWM) insulated-gate bipolar transistor (IGBT) VSI located at the load side. The solar PV system consists of PV array and DC-DC converter with maximum power point tracking (MPPT) algorithm. In PV system, MPPT is used to increase the system efficiency by controlling DC-DC converter. The OWC system was configured by the bidirectional Darrieus turbine driven permanent magnet synchronous generator (PMSG) and an AC-DC three-phase rectifier.

In the HRES, the renewable PV and wave energy system is considered as a main power generation source to meet the system load demand and battery bank is used as a backup energy storage system. The HRES is proposed to implement in island areas in Malaysia; hence, if generated power from HRES is not enough to meet the system load demands, then battery bank will deliver power to balance the system power demand. To interface PV, wave, and battery bank in hybrid framework, the dc-link voltage must be constant. Hence, a BBDC with PI controller is used in the HRES to maintain the constant dc-link voltage. A three-phase VSI with relatively complex vector control scheme is used at load side to control load side voltage in terms of the amplitude and frequency. The detailed description of each component of the overall HRES and controller is given in the following parts.

### 2.1. Modeling and Characteristics of PV System

**Figure 2:**
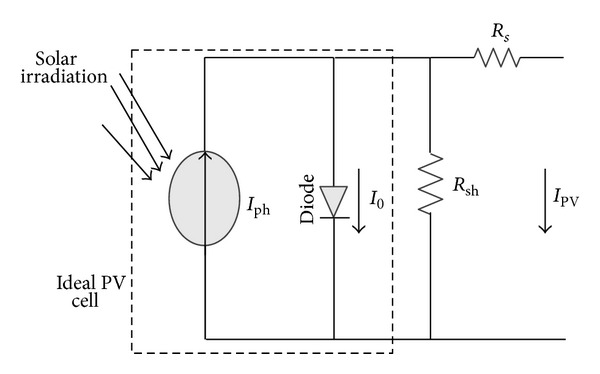
Circuit diagram of single diode PV model.

Solar PV systems generate electric power by converting solar photon energy into electrical energy in the form of direct current using solar cell or PV cell. Crystalline or polycrystalline materials are commonly used for solar cell [17]. Each of the PV cells produces around 0.5 V and it is the smallest unit of the solar PV system. Cells are further connected in series or/and parallel combination to form a PV array.  Figure 2 shows widely used one diode equivalent circuit model for a PV cell [18]. PV cell equivalent circuit model consists of a current source parallel with a diode and the output terminals of the circuit are connected to the load through the shunt and series resistor. The current-voltage characteristics of PV array can be expressed using some nonlinear mathematical exponential equations. The ideal relationship between voltage and current is given by [18–21]

(1) IPV=Iph−ID−Ish=Iph−I0[exp⁡qAKT(VPV+IPVRs)−1] −VPV+IPVRsRsh,

where *I*_PV_ is the output current of the PV cell (A), *I*_ph_ is the photocurrent, *I*_*D*_ is the diode current, *I*_sh_ is the current through the shunt resistance, *I*_0_ is the reverse saturation current, *K*, the Boltzmann constant = 1.38 × 10^−23^(J/K), *q*, the charge of electron = 1.6 × 10^−19^ (*C*), *T* is the cell temperature (K), *V*_PV_ is the output terminal voltage of the PV cell (V), *A* is the quality factor (lies between 1.2 and 1.6 for crystalline silicon), *R*_*s*_ is the series resistance (Ω), and *R*_sh_ is the shunt resistance (Ω).

**Figure 3:**
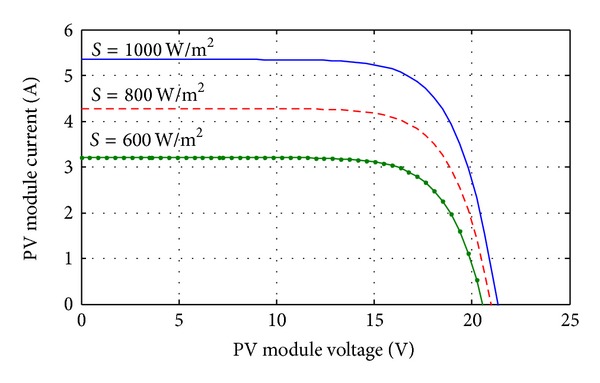
KOYCERA KC85T-87W PV model I-V characteristics curve with varying irradiation.

**Figure 4:**
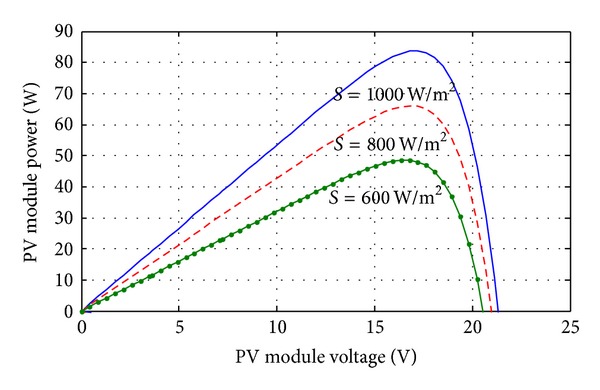
KOYCERA KC85T-87W PV model P-V characteristics curve with varying irradiation.

The output power from solar PV array is given by

(2) $$\begin{array}{c} P_{\text{PV}}=V_{\text{PV}}I_{\text{PV}}\eta_{\text{conv}}, \end{array}$$

where *η*_conv_ is the DC-DC converter efficiency (typically 90–95%). In this paper total five KOYCERA KC85T-87W PV models are used for 400 W power generation and all the five models connected in series with one another. The power-voltage and current-voltage characteristics of KOYCERA KC85T-87W PV model are obtained according to the value of the variables *I*_ph_, *I*_0_, *R*_sh_, and *R*_*s*_. The value of the variables can be collected from [18]; they usually provide values for *I*_PV_ and *V*_PV_ at open circuit, short circuit, and maximum power point and finally the number of the PV cells. The current-voltage and power-voltage characteristics of a solar PV module operating at a standard temperature of 25°C and different solar irradiance are shown in Figures 3 and 4.

**Figure 5:**
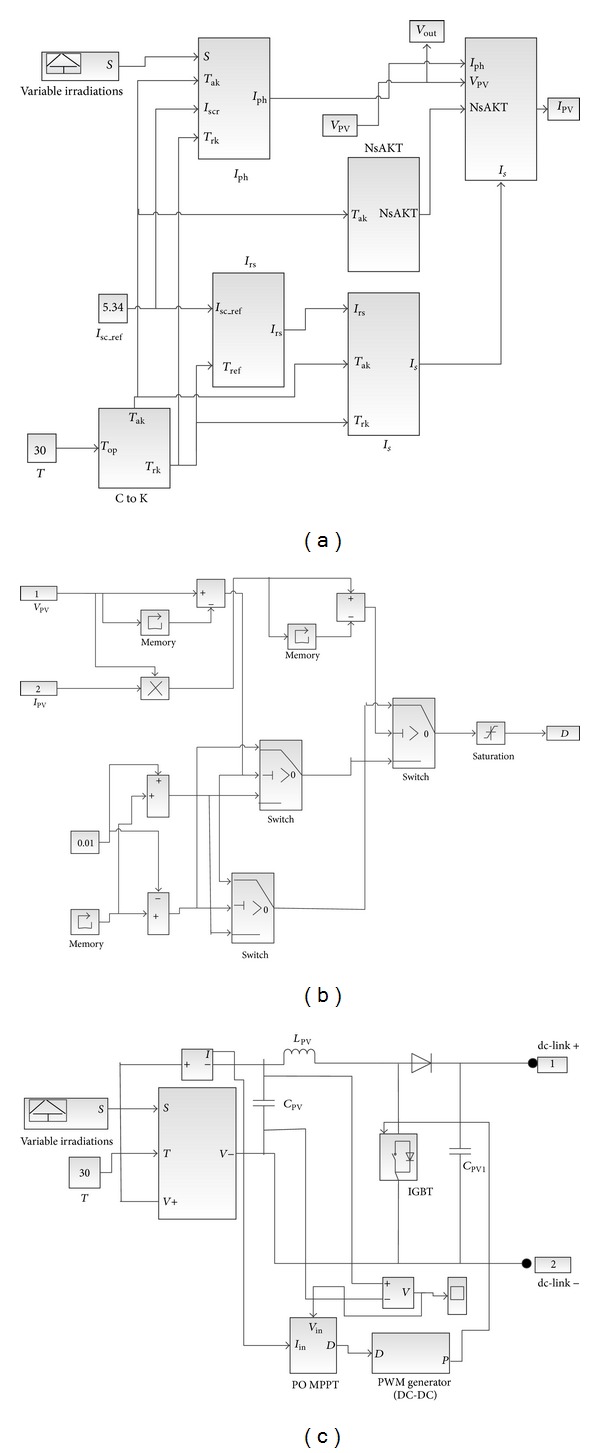
The Simulink diagram of the PV model with MPPT. (a) PV Simulink model; (b) MPPT model; (c) complete Simulink PV model with MPPT.

According to solar irradiation or load current, the maximum output power of the PV module varies. Therefore, a proper control system is needed to use the PV model more efficiently as an electric power source by building a MPPT. There are many different MPPT methods discussed in [18, 19, 22], among them perturbation and observation method (P&O) is most widely used because it is much simpler and needs fewer measured variables. In this paper from [19], P&O method is used for building MPPT in Simulink environment. According to (1), (2), and the literature described in [20, 21], PV model with MPPT is developed using Matlab Simulink, which is illustrated in Figures 5(a), 5(b), and 5(c).

### 2.2. Modeling of OWC

It should be noted that this paper focuses on the designing of a battery storage standalone PV-wave hybrid supply system for island communities and, therefore, the mathematical modeling for individual elements such as OWC wave chambers is simplified. The detailed design and complete mathematical modeling of OWC wave energy system can be found in [23–29], where more precise model is established.

**Figure 6:**
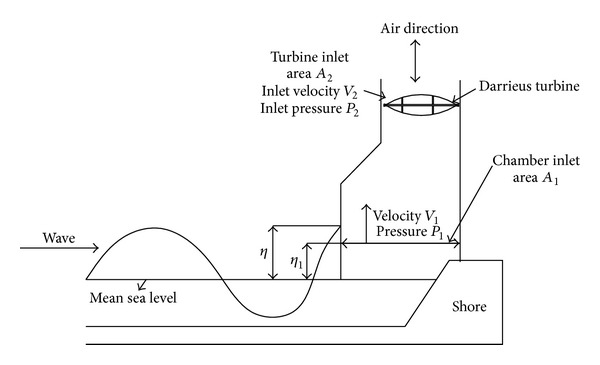
OWC chamber parameters.

The operating principle of the OWC as shown in Figure 6 is much like a wind energy system via the wave induced air pressurization principle. In this system sea wave motion causes the rise and fall of the water level within the wave chamber. This causes pressure oscillations, which can be used to drive a bidirectional air turbine. The bidirectional turbine extracts the kinetic energy of sea wave and turned it into mechanical energy which is fed into the electrical generator. The generator converts this mechanical energy into electrical energy which will feed directly either to the load or in the grid.

In this section, a set of equations is present to describe the power generated by OWC system. As mentioned earlier, OWC wave energy operating principle is much like wind turbine system, so the power available at the wave turbine consists of two terms: air velocity term *P*_*a*_ and air pressure term *P*_pt_. Therefore, the total inlet power can be described using the following equation:

(3) $$\begin{array}{c} \text{Inlet}\;\text{Power}\;P_{\text{in}}=P_{a}+P_{\text{pt}}, \end{array}$$

where the power *P*_*a*_ acting on the turbine due to the air velocity term is

(4) $$\begin{array}{c} P_{a}=\frac{\rho A_{2}V_{2}^{3}}{2}. \end{array}$$

The available output power developed by the OWC is a function of the turbine power coefficient *C*_*P*_, so the total output power developed by the OWC is

(5) $$\begin{array}{c} P_{\text{total}}=\left(P_{a}+P_{\text{pt}}\right)\times C_{P}. \end{array}$$

The power due to the air velocity term is straightforward and shown in (4). But power due to the pressure term is more complex. It is mainly depending on the surface elevation in OWC chamber. But this wave surface elevation further depends on the two major factors, namely, the turbine inlet velocity and air pressure term. Both of these factors are related to the OWC chamber length, water depth, and so forth. So, in this paper, the mathematical modelling of OWC wave energy system mainly focuses on the air pressure term and its derivative from the literature [23–29], which is discussed here.

**Figure 7:**
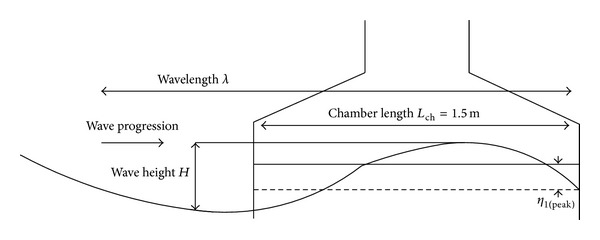
OWC full chamber arrangements [23].

Figures 6 and 7 illustrate the parameters related to the OWC. At first, it assumed that the regular outer wave free surface elevation can be stated as [24]

(6) $$\begin{array}{c} \text{Outer}\;\text{wave}\;\text{surface}\;\text{elevation}\;\eta_{0}=\frac{H}{2}\cos\left(\frac{2\pi }{T}t\right), \end{array}$$

where *H* is the wave height (m) and *T* is the wave period (s). If the chamber length of OWC is *L*_ch_ with respect to the wavelength, then free surface elevation in OWC can be approximated as follows:

(7) $$\begin{array}{c} \eta_{1}=\frac{H_{\text{in}}}{2}\cos\left(\frac{2\pi }{T}t\right)\times \frac{2\sin\left(\theta /2\right)}{\theta }, \end{array}$$

where *H*_in_ is the averaged internal wave height (m) and it is calculated from the literature in [23, 25]. The angular chamber length *θ*(rad) is defined as

(8) $$\begin{array}{c} \theta =\frac{2\pi L_{\text{ch}}}{\lambda }, \end{array}$$

where *λ* is the actual wave length; for calculating this wavelength an equation can be formulated [30] as follows:

(9) $$\begin{array}{c} \lambda ≅\left(1-\frac{\pi d}{3\lambda_{0}}\right)\times T\sqrt{gd}, \end{array}$$

where *d* is the water depth (m), *g* is the gravitational constant (9.81 ms^−2^), and *λ*_0_ is the theoretical deep water wavelength, which is given by [23]

(10) $$\begin{array}{c} \lambda_{0}=\frac{gT^{2}}{2\pi }. \end{array}$$

The velocity of the air adjacent to the internal free surface is the liner velocity of water height, where

(11) $$\begin{array}{c} V_{1}=\frac{d\eta_{1}}{dt}=-\frac{\omega H_{\text{in}}}{\theta }\sin\left(\omega t\right)\times \sin\left(\frac{\theta }{2}\right),\\ \omega =\frac{2\pi }{T}. \end{array}$$

Since the system is relatively low-pressure system, so the axial velocity passage through the turbine is

(12) $$\begin{array}{c} V_{2}=\frac{A_{1}}{A_{2}}V_{1}=-\frac{A_{1}}{A_{2}}\frac{\omega H_{\text{in}}}{\theta }\sin\left(\omega t\right)\times \sin\left(\frac{\theta }{2}\right), \end{array}$$

where *A*_1_ is the flow surface area of the chamber and *A*_2_ is the inlet turbine area. The power *P*_pt_ available at the turbine depends on the volume of air flow rate *Q* across the turbine and the gradient of pressure. Hence, the power available at the turbine due to the air pressure term (for completeness, this equation is derived in Appendix D) is

(13) Ppt=[−A1A2Hin2θ2ω2{2cos⁡⁡(ωt)2−1}} × sin2(θ2)+QA2(V2−V1)]×Q×ρ.

**Figure 8:**
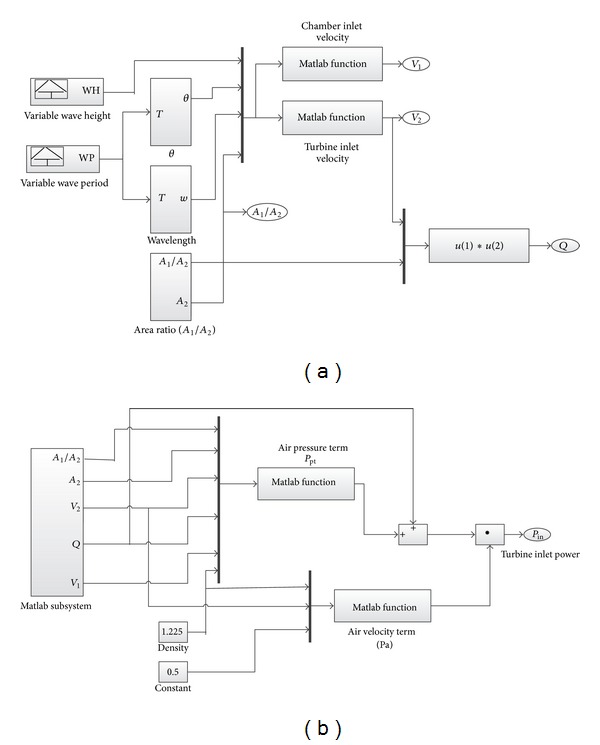
The Simulink diagram of the OWC model.

According to (3) and (13), OWC model is developed using Matlab Simulink, which is illustrated in Figures 8(a) and 8(b).

### 2.3. Modeling of Battery

A standard battery model presented in [31] is implemented in this paper. To avoid the battery algebraic loop problem, this model uses only the state of charge (SOC) of the battery as a state variable. Moreover, model in [31] can precisely characterize four types of battery chemistries including lead-acid battery.

**Figure 9:**
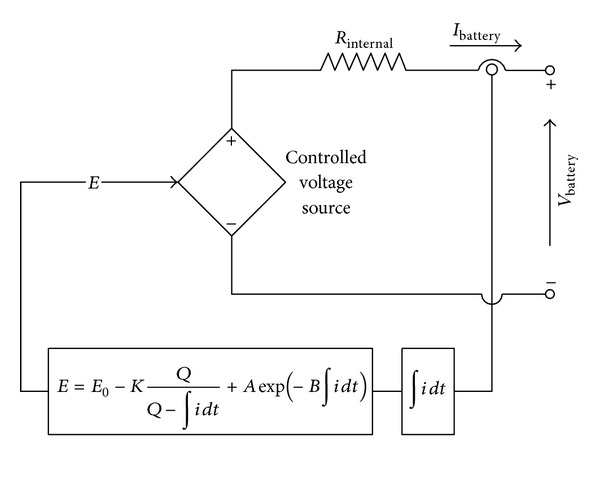
Nonlinear standard battery model [31].

The battery is modeled using a simple series connected controlled voltage source with a constant resistive value, as shown in Figure 9, where the controlled voltage source is described by

(14) $$\begin{array}{c} E=E_{0}-K\frac{Q}{Q-\int i\;dt}+A\exp\left(-B\int i\;dt\right), \end{array}$$

(15) $$\begin{array}{c} V_{\text{Battery}}=E-R_{\text{in}}I_{\text{Battery}}, \end{array}$$

where *E*_0_ is the no load battery voltage (V), *K* is the polarization voltage (V), *Q* is the battery capacity (Ah), *A* is the exponential zone amplitude (V), *B* is the exponential zone time constant inverse (Ah)^−1^, *V*_Battery_ is the battery voltage (V), *R*_in_ is the battery internal resistance (Ω), *I*_Battery_ is the battery current (A), and ∫*i* *dt* is the charge supplied and drawn by the battery (Ah).

**Figure 10:**
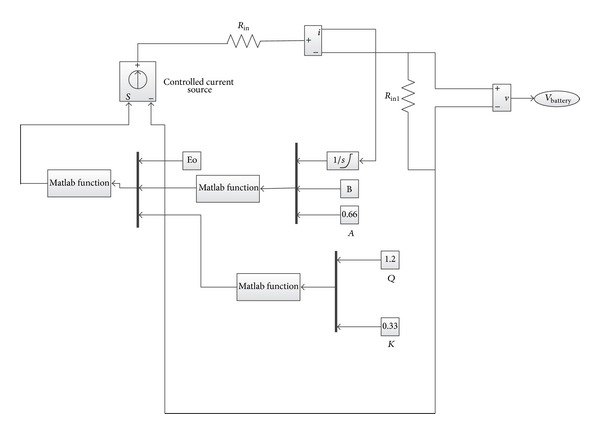
The Simulink diagram of the battery model.

The battery model based on (14) is developed in Matlab Simulink environment and connected to a DC-DC buck-boost bidirectional converter using controlled voltage source as shown in Figure 10.

### 2.4. Control of dc-Link Voltage

**Figure 11:**
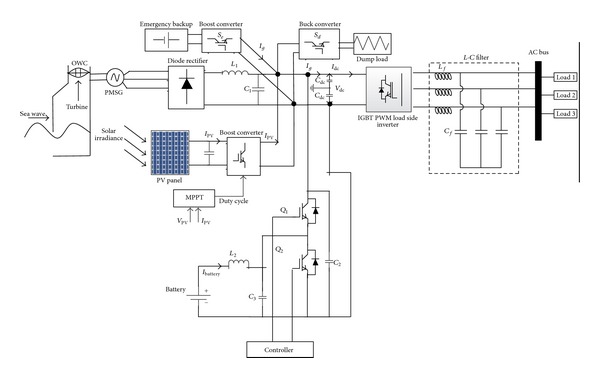
Circuit topology of the proposed PV-wave hybrid standalone system with emergency backup and dump load.

The circuit topology of the proposed PV-wave hybrid standalone system is shown in Figure 11. A neutral wire is placed between the capacitors connected before the VSI for feeding single-phase as well as three-phase loads to the proposed system, as shown in Figure 11.

**Figure 12:**
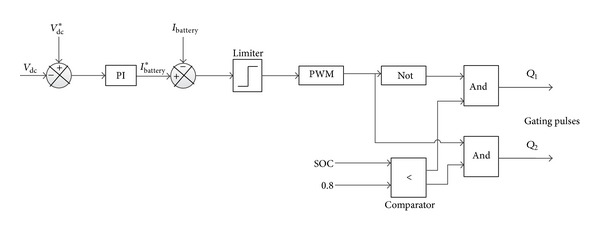
The schematic diagram of DC-DC converter controller.

In this paper, through BBDC the dc link side is connected to batteries bank; the primary objective of the control of this BBDC is to maintain constant dc-link voltage as a reference value in addition to discharge/charge current from/to batteries bank according to the required load power. The schematic diagram of the battery bank BBDC controller is depicted in Figure 12. The voltage of the battery bank can be kept lower as compared to the reference dc-link voltage (*V*_dc_*) by using BBDC and hence fewer numbers of batteries are required to be connected in series. In the proposed standalone system, the voltage of the battery bank is kept at around 300 V, whereas *V*_dc_* = 650 V. In this paper, the batteries bank depth of discharge is considered 60% [13] and is based on the assumption that it should provide the electric power to the loads of a 2.5 kW for approximately an hour when the generated wave power is zero. The details of the rating of the batteries bank calculation are discussed in Appendix A.

The value of the inductor used in BBDC is crucial for the conduction mode operation of it. And also the inductor existence in the batteries bank side is shown lower ripple current results which gives long lifetime and higher efficiency. Conduction mode operation also depends on input and output current, capacitors value, and switching frequency. The value of the inductor and capacitors is as follows [32, 33]:

(16) $$\begin{array}{c} \text{Inductance}\;L_{2}=\frac{V_{\text{Battery}}\times \left(V_{\text{dclink}}-V_{\text{Battery}}\right)}{I_{\text{Battery}}\times f_{s}\times V_{\text{dclink}}},\\ \text{Buck}\;\;\text{mode}\;\;\text{capacitance}\;\;C_{2}=\frac{k_{L}\times I_{\text{Battery}}}{8\times f_{s}\times V_{\text{Battery}(\text{ripple})}},\\ \text{Boost}\;\;\text{mode}\;\;\text{capacitance}\;\;C_{3}=\frac{D_{\text{Boost}}\times I_{\text{dclink}}}{f_{s}\times V_{\text{dclink}(\text{ripple})}}, \end{array}$$

where *V*_Battery_ is the battery bank voltage, *V*_dclink_ is the dc-link voltage, *I*_dclink_ is the dc-link current, *I*_Battery_ is the battery bank current, *V*_Battery(ripple)_ is the buck side output desired ripple voltage, *V*_dclink(ripple)_ is the boost side output desired ripple voltage, *k*_*L*_ is the estimated coefficient of indicator ripple current at buck side, and *f*_*s*_ is the switching frequency.

**Figure 13:**
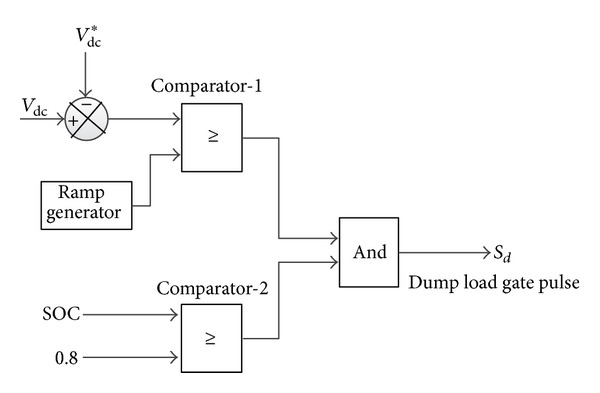
The schematic diagram of dump load controller.

In this paper, the battery bank can act either as a power supply or as a sink. As a result, it should discharge/charge within specified limits when there is lack/surplus of hybrid power due to the weather condition. In this work, due to high wave and solar power condition, the surplus power at first is supplied to the battery bank until it reach its upper limit of charge carrying capacity and then additional power is absorbed by the dump load and is regulated via the chopper control shown in Figure 13. In this case, controller switching decision is made by comparing the upper limit of SOC and present status of SOC.

**Figure 14:**
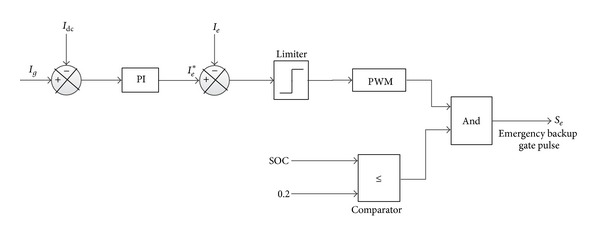
The schematic diagram of emergency backup controller.

In case of long term when there is no PV and (/or) wave or lower PV and (/or) wave power the battery bank may not able to meet the load demand. In this case, an emergency backup is integrated with hybrid system. Control action algorithm of emergency backup is depicted in Figure 14.

**Figure 15:**
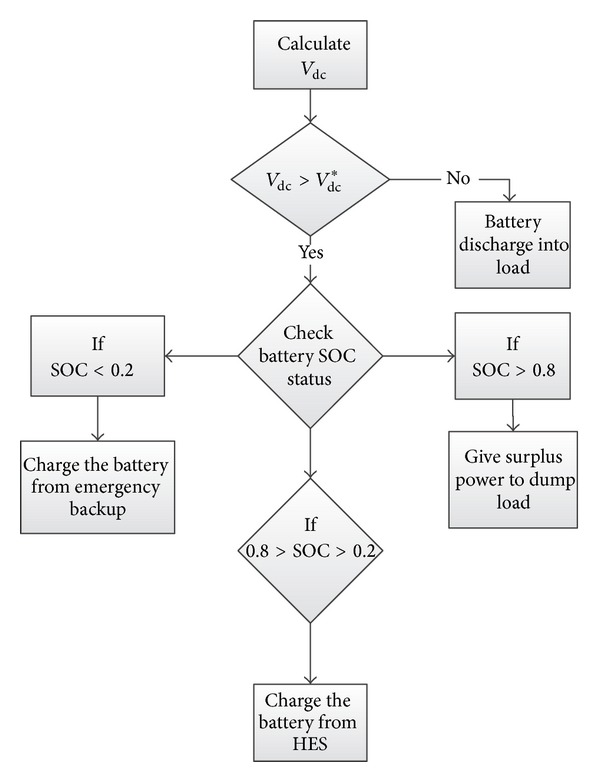
Flowchart for dc-link voltage control.

Figure 15 shows a dc-link voltage control flowchart based on the above discussion for controlling the dc-link voltage, where the lower and upper limits for the battery bank SOC are kept at 0.2 and 0.8, respectively [13].

### 2.5. Control of Load Side VSI

At the load end, a three-phase vector control VSI is used as interface element between the consumer load and DC link voltage. The load side VSI control is responsible to control the frequency and voltage at the consumer load end. In the proposed HRES system, the output load voltages should be controlled in terms of frequency and voltage amplitude because there is no electric power grid connection. The space vector control technique is used to regulate the output voltage during the variation of required hybrid power or load power.

In this paper, the vector control technique is used based on the synchronously rotating frame described in [34, 35]. The three-phase *V*_*a*_, *V*_*b*_, *V*_*c*_ voltages and *I*_*a*_, *I*_*b*_, *I*_*c*_ currents should be transformed and measured from the reference stationary *a*-*b*-*c* frame to the reference rotating *d*-*q* frame using the preferred output load voltage electrical frequency. In this paper, the specified root-mean-square (RMS) valuee of the output phase voltage and the load voltage frequency are 220 V and 50 HZ, respectively.

The equations of voltage using reference rotating *d*-*q* frame transformation are taken from [34, 35] as follows:

(17) $$\begin{array}{c} v_{d}=v_{di}-L_{f}\frac{di_{d}}{dt}+L_{f}\omega i_{q},\\ v_{q}=v_{qi}-L_{f}\frac{di_{q}}{dt}-L_{f}\omega i_{d}. \end{array}$$

By using *d*-*q* reference rotating frame transformation, the active power and reactive power are given by

(18) $$\begin{array}{c} \text{Active}\;\text{power}\;\;P=\frac{3}{2}\left(v_{d}i_{d}+v_{q}i_{q}\right),\\ \text{Reactive}\;\text{Power}\;\;Q=\frac{3}{2}\left(v_{d}i_{q}+v_{q}i_{d}\right). \end{array}$$

The active and reactive power equations will be as follows if the reference rotating frame is as *v*_*q*_ = 0 and *v*_*d*_ = |*V*|:

(19) $$\begin{array}{c} P=\frac{3}{2}v_{d}i_{d}=\frac{3}{2}\left|V\right|i_{d},\\ Q=\frac{3}{2}v_{d}i_{q}=\frac{3}{2}\left|V\right|i_{q}. \end{array}$$

**Figure 16:**
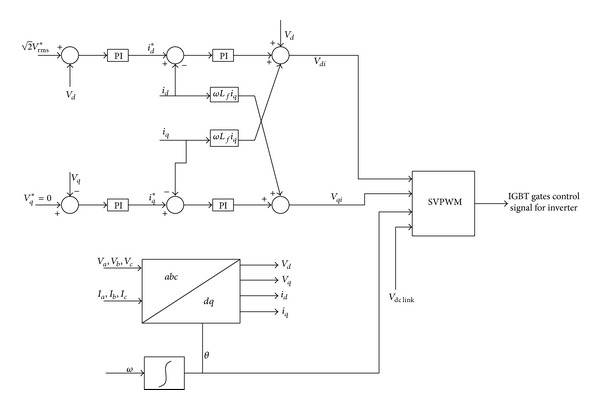
The load side three-phase VSI controller.

Therefore, the active and reactive power can be controlled by controlling direct and quadrature current elements, respectively. Also, for resistive load, case *V*_d_* can be directed by

(20) $$\begin{array}{c} V_{d}^{*}=\sqrt{2}V_{\text{RMS}}^{*}, \end{array}$$

where *V*_RMS_* is the output RMS phase voltage reference value. In this control technique, the load output current in the internal control loops and the load output voltages in the external control loops are regulated by PI controllers. All the PI controllers used in this paper are tuned by using Ziegler-Nichols tuning method [36]. The control technique of the VSI is used in load side as shown in Figure 16.

Based on the inverter switching frequency, the high frequency unwanted harmonics will generate in output ac voltage by the load side VSI which ultimately creates power quality problem in the customer end. In this controller, space vector PWM (SV-PWM) method is used because it slightly reduces the system harmonics contents in the output voltage. In addition, it raises the fundamental load output voltage. A simple passive *L*-*C* filter is used in load side end to eliminate the high frequency unwanted harmonics for preventing the power quality problem occurring in the customer end. The design of the passive *L*-*C* filter [37] is given in Appendix B and the values are

(21) $$\begin{array}{c} L_{f}=0.052\;\text{H},\\ C_{f}=2\;\mu \text{F}. \end{array}$$

## 3. Site Selection

**Figure 17:**
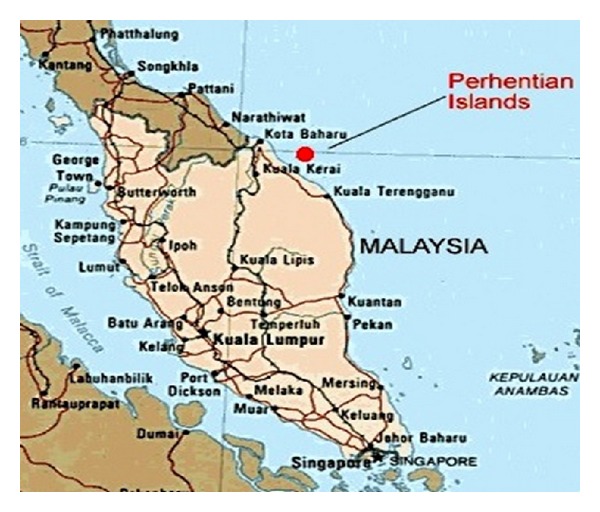
Location of target site (Perhentian Island) (modified from [45]).

Malaysia is situated between 1° and 7° in the North Latitude and 100° and 120° in the East Longitude. But Malaysia is vastly surrounded by water and it has the 29th longest coastline in the world. It has a total coastline of 4,675 kilometers and 878 islands [38, 39], so Malaysia has a massive potential of wave energy that may be a vital source of electrical energy generation especially for the coast and the island communities. Among those islands, Perhentian Island (shown in Figure 17) is one of the most popular resort islands in Malaysia situated in the northeastern coast of Western Malaysia. It is approximately 20 km from the coast of Terengganu. In this island, the majority of the buildings are resorts and only one village where the local people live. Diesel generators are used as the main sources for electricity generation in this island. In 2007 [40], Universiti Kebangsaan Malaysia and National Energy Policies (NEP) installed a wind-solar hybrid energy system in this island, although till now it was not connected to the electrical network because of lower wind power. The winds are rare in this area.

**Figure 18:**
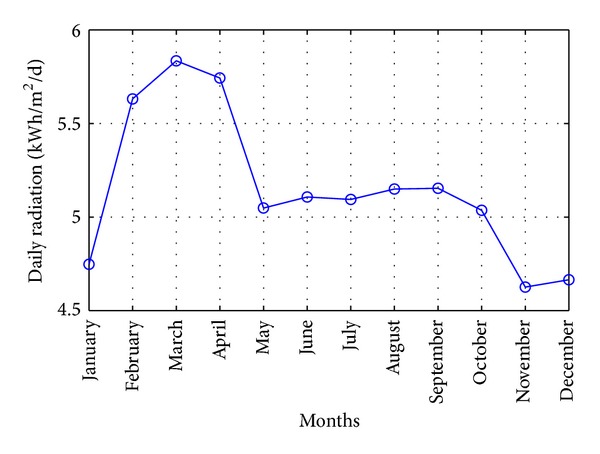
Annually solar irradiation.

Perhentian Island has uniform climate characteristics and it has abundant rainfall and temperature with high humidity. Since it is located in the equatorial doldrums area, it has naturally great sunshine and solar radiation. But it is quite difficult to have a fully sunny day with completely clear sky. Perhentian Island receives daily average 5.5 hours of the sunshine. The solar data of Perhentian Island is as shown in Figure 18.

**Figure 19:**
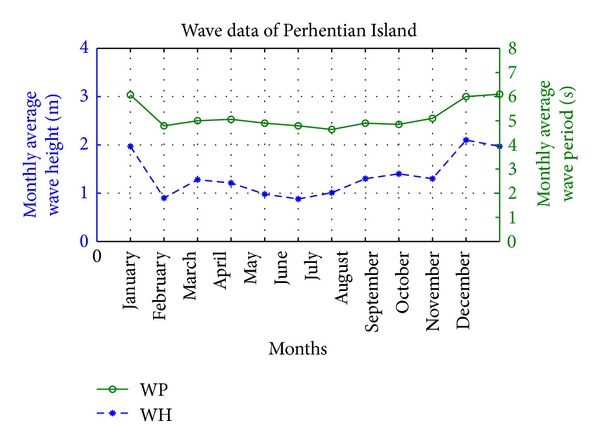
Monthly average WH/WP data of a year for Perhentian Island.

Solar energy cannot meet the consumer load demand at each instant because of its daily limited sunshine hours. So to build HRES in this island, wave energy can be considered one of the efficient power generation sources. The wave data of Perhentian Island is illustrated in Figure 19, where the peak average wave height (WH) and wave period (WP) occur from November to January. Both solar and wave data are obtained by Malaysian Meteorological Department Labuan (MMDL) from 2005 to 2012. In addition, wave data is analyzed by the “hindcast” technique [41]. MMDL collected wave data by using Acoustic Doppler Current Profiler (ADCP) equipment and Voluntary Observation Ship (VOS) scheme.

**Figure 20:**
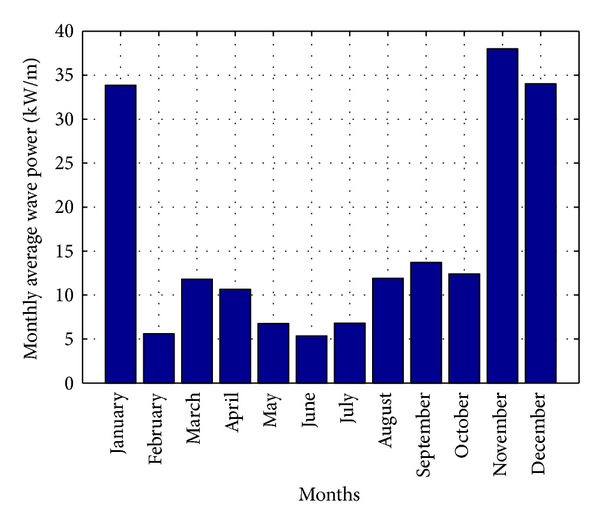
Average wave power level of a year for Perhentian Island.

At this site, the maximum average WH measured in the month of November was 2.1 m and the minimum in the month of June was 0.88 m. On the other hand, the maximum average WP measured in the month of December was 6.1 s and the minimum in the month of July was 4.64 s. Based on the wave theory and equations in [1, 29], wave power level during the whole year is shown in Figure 20 and is mainly depending on WH and WP.

It could be found from [1] that Malaysian sea has an average of 8.5 kW/m wave power level. But, from Figure 20, it could be seen that Perhentian Island has an average of 15.9 kW/m wave power level. So Perhentian Island site is identified as economically viable for commercial scale wave power generation in Malaysia, because any site in the world is able to produce wave power at competitive prices if it has an average wave power level equal or above 15 kW/m. In addition, these sites are considered to have exceptionally high-energy resources than other renewable energy sources like wind. The main aim of the propose hybrid system in this paper is to establish a commercial scale PV-wave hybrid power plant in the Perhentian Island by eliminating the intermittent power generation nature of both PV and wave energy sources.

## 4. Simulation Result and Discussion

The simulation model of the proposed standalone PV-wave hybrid system with energy storage is built in Matlab Simulink environment under different operating conditions. PMSG is modeled in Matlab Simulink from the literature [42, 43] and the parameters are taken from [44] which are presented in Appendix C. In addition, the parameters used for PV array, OWC design, and Darrieus turbine are also mentioned in Appendix C. In this section, the average solar irradiation of January, February, and March months and the average wave height and wave period of February, March, and June months from Section 3 are used to observe the performance of the proposed system under the variable load condition.

**Figure 21:**
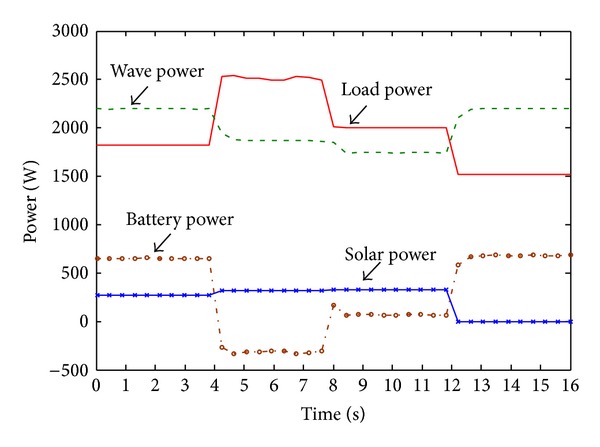
PV-wave hybrid system powers distribution.

**Figure 22:**
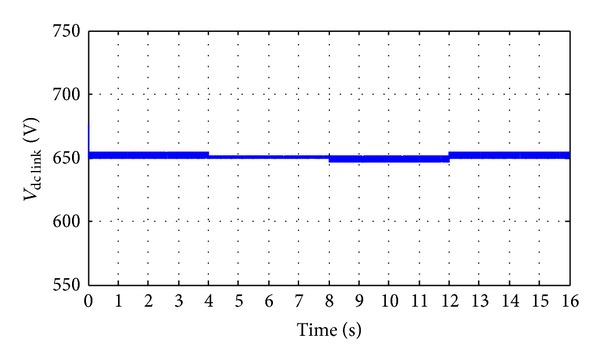
Dc-link voltage.

The performance of buck-boost DC-DC bidirectional converter controller is presented in Figures 21 and 22.  Figure 21 shows the power distribution curve of generated solar power, wave power, load power, and battery bank power. From Figure 21 it is seen that during the solar power, wave power, and required load variation, power from the battery bank changes (discharge/charge) to maintain the power stability of the system. So it could be clear from Figure 21 that, when the generated hybrid power is more than the required load power, the controller is able to charge the battery bank, and when the required load power is more than generated hybrid power then controller is able to discharge the battery bank. Further, it can also establish the constant dc-link voltage at 650 V when there is a change in hybrid power and load demand, as shown in Figure 22. The absence of solar power after 12-second in Figure 21 indicate that cloudy or night period. In this case, wave power and battery bank power satisfactorily meet the load demand at each instant. So it can be established that the performance of the buck-boost DC-DC bidirectional converter controller is quite satisfactory in both transient and steady-state hybrid power and load demand condition.

**Figure 23:**
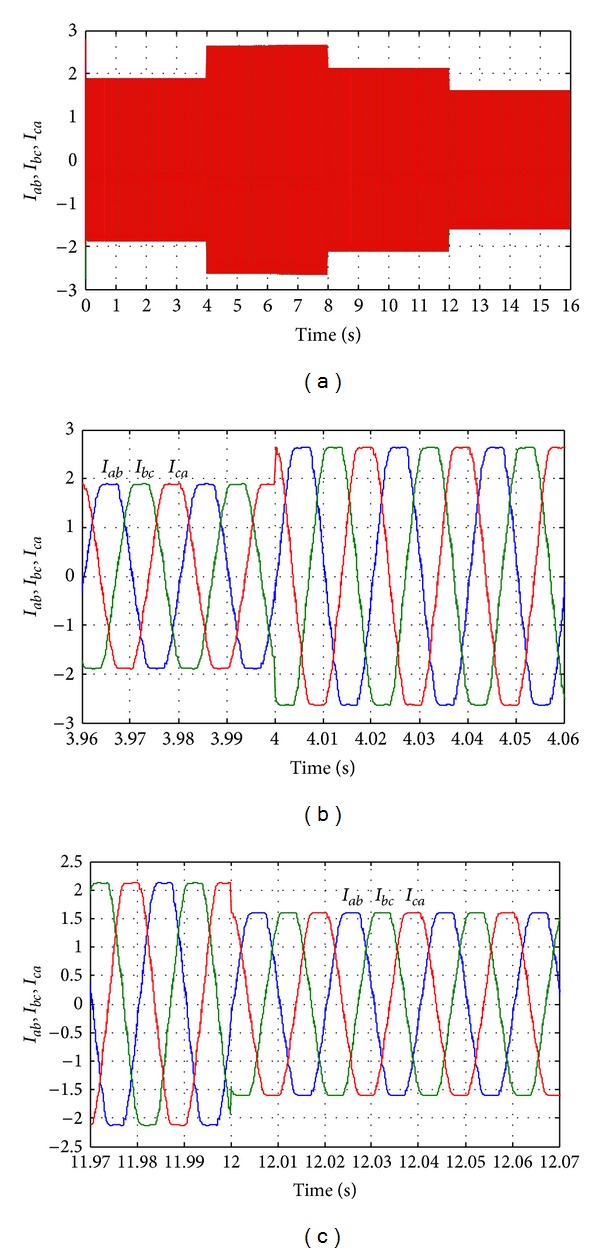
Output line current response with change in required load power. (a) Output line currents throughout the full simulation time; (b) output line current when the load increases at simulation time from 3.96 s to 4.06 s; (c) output line current when the load decreases at simulation time from 11.97 s to 12.07 s.

**Figure 24:**
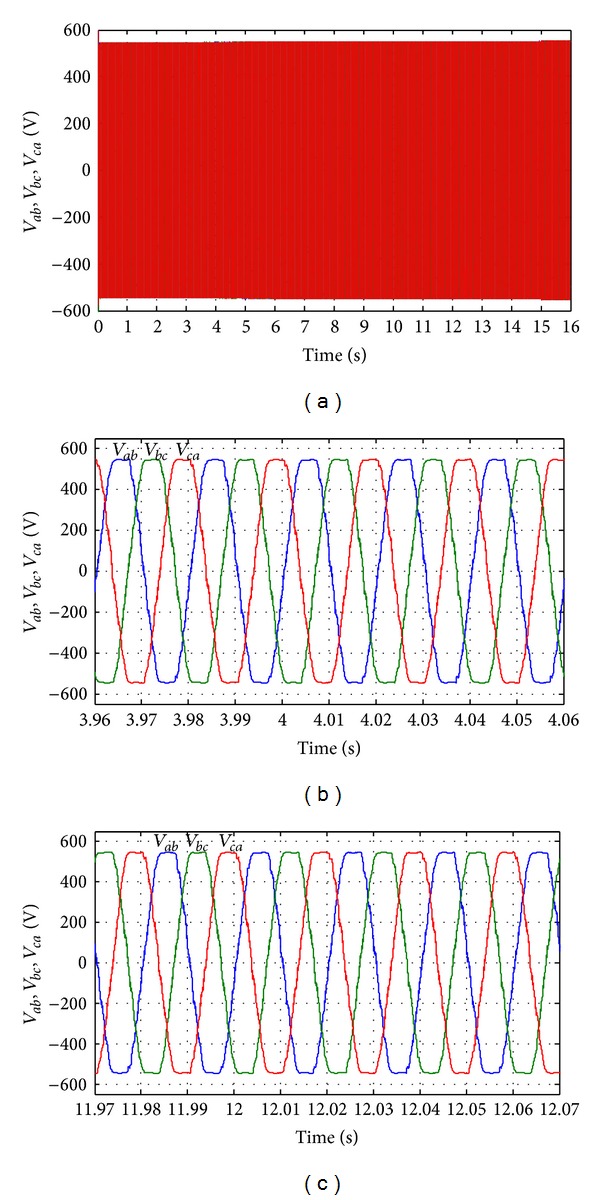
Output line voltage response with change in required load power. (a) Output line voltages throughout the full simulation time; (b) output line voltage when the load increases at simulation time from 3.96 s to 4.06 s; (c) output line voltage when the load decreases at simulation time from 11.97 s to 12.07 s.

The output load current response during the long time simulation is shown in Figure 23(a). Figures 23(b) and 23(c) show the output load current when the load power increases at simulation time from 3.96 s to 4.06 s and when the load power decreases at simulation time from 11.97 s to 12.07 s, respectively. The output voltage response for whole simulation time and the output voltage response when load increases and decreases are shown in Figures 24(a)–24(c), respectively.  Figure 24 shows the output RMS phase voltage (for phase a *V*_*a*_) where it is maintained 220 V as reference value. In Figures 26(a) and 26(b), it is seen that the total harmonic distortion (THD) in the output ac line voltage is about 1.8% and similarly the THD in the output ac line current is about 1.5% in all the three phases. The modulation indexes are shown in Figure 27 for all the three phases.

**Figure 25:**
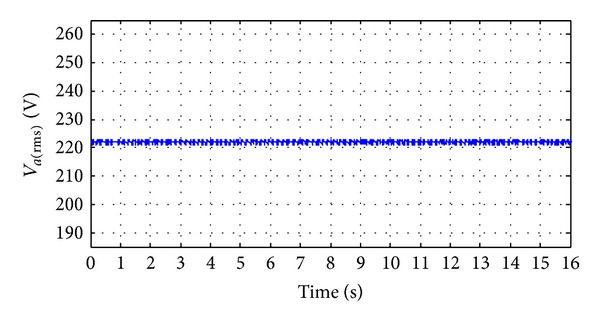
The output RMS phase voltage.

**Figure 26:**
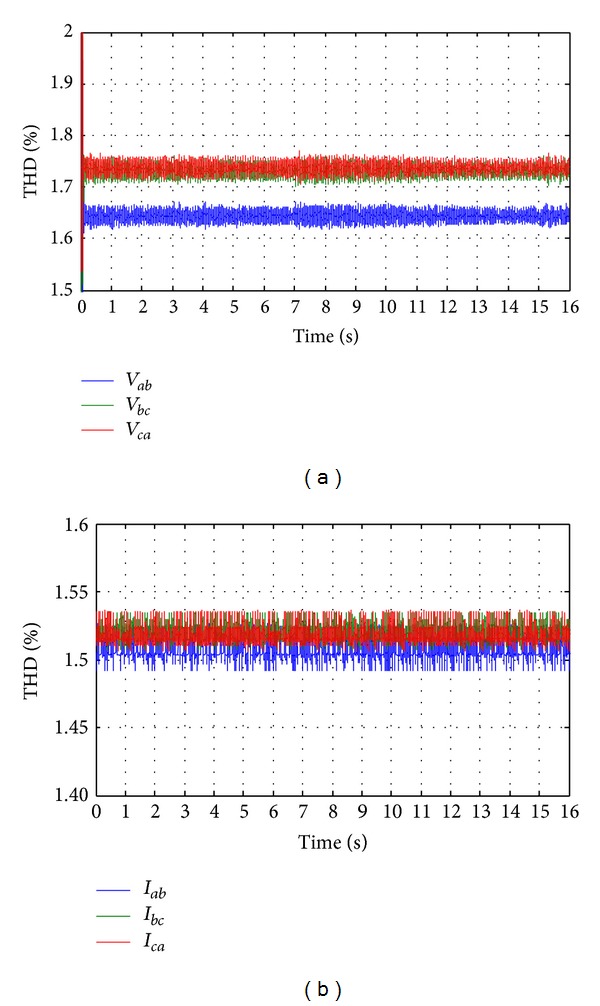
The total harmonic distortion throughout the whole simulation time. (a) THD of line voltage; (b) THD of line current.

**Figure 27:**
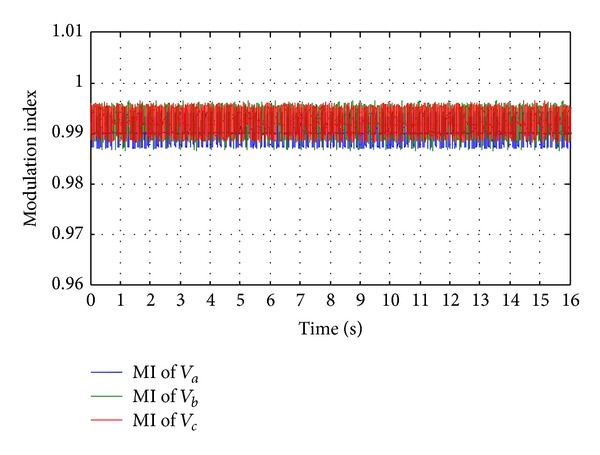
Modulation indexes for all three phases.

From Figures 24, 25, 26, and 27, it can be clear that a suitable quality of voltage and current can be delivered to the load with the help of SV-PWM control inverter switching and a passive *L*-*C* filter. Finally, it ascertained that the proposed hybrid system can successfully accommodate solar irradiation, WH, WP, and load changes, and the control system can efficiently track the change of hybrid power generation and load demand.

## 5. Conclusion

In this paper, a novel standalone PV-wave hybrid system with appropriate energy flow controllers is designed and modeled for island users where the electric power grid is not available. The power generated by PV sources is highly dependent on environmental conditions. To overcome this intermittent power generation nature of PV system, in this paper, PV system integrated with the wave energy converter device and battery bank, because wave energy is easily predictable and consistent than other green energy sources such as wind. The hybrid PV-wave topology with broad analysis and Matlab Simulink simulation results is presented in this paper. It has been seen from the simulation results that the controller can maintain the dc-link voltage at constant value in spite of variation in generated hybrid power and required load power. Furthermore, the controller is developed in such a way that the battery bank has been able to accumulate the excess power generated by hybrid system and supply it to the system load during the hybrid power shortage by controlling BBDC. This controller not only maintains the constant dc-link voltage, but also acts as a dc-link side active filter and reduces the generator toque oscillation of OWC PMSG during the variation in load. Finally, it has been explained how the space vector controls three-phase VSI controller to control the load side output voltage in terms of frequency and voltage amplitude to the resistive load. The THD in voltage and current at load side is about 1.7% and 1.55%, respectively, which illustrates the good quality of voltage and current generated at the consumer side end. The simulation results show that the performance of the proposed hybrid system is satisfactory under the steady-state power as well as transient solar, wave, and load power conditions. This study can be considered as the initial part of building prototype standalone PV-wave hybrid system. The future work will aim to set up a standalone PV-wave hybrid system in the University of Malaya laboratory to verify the simulations results with experiment.

## Figures and Tables

**Table 1:** Parameters of PMSG.

Number of poles	4
Rated power	3 kW
Rated speed	241 rad/s
Per phase stator resistance (*R*)	0.4578 Ω
*d*-axis and *q*-axis stator inductance (*L*_*d*_ & *L*_*q*_)	0.00334 H
Magnetic flux induced in the stator windings (*ψ*)	0.171 Wb
Rated torque	14.2 Nm

**Table 2:** Parameters of OWC.

OWC chamber length (*L*_ch_)	1.5 m		
Water surface area inside the chamber (*A*_1_)	1.4 m^2^		
Turbine inlet area (*A*_2_)	0.012 m^2^		
Water depth (*d*)			
WH (m)	0.98	0.9	0.88
WP (s)	4.9	4.79	4.79
*d* (m)	16.47	15.75	15.73

**Table 3:** Parameters of Darrieus turbine.

Swept area by balde (*A*)	0.012 m^2^
Air density	1.22 Kg/m^3^
Height of the rotor (*H*_*t*_)	240 mm
Diameter of the rotor (*D*_*t*_)	100 mm

**Table 4:** Parameters of PV array.

Maximum rated power (*P*_max⁡_)	87 W
Maximum voltage (*V*_max⁡_)	17.4 V
Maximum current (*I*_max⁡_)	5.02 A
Open circuit voltage (*V*_oc_)	21.7 V
Short circuit voltage (*I*_sc_)	5.34 A
Number of modules required	5

## Appendices

### A.

The battery bank rating calculation is

(A.1) $$\begin{array}{c} \text{Battery}\;\text{rating}\;=\frac{2.5\;\text{kW}\times 1\;\text{hr}}{300\;\text{V}\times 0.6}=13.89\;\text{Ahr}. \end{array}$$

Hence, 12-V, 14-Ahr battery rating is considered and, consequently, 25 numbers of batteries are required to connect in series.

### B.

The equations [13, 37] related to passive *L*-*C* filter design are

(B.1) $$\begin{array}{c} K={\left[{\frac{k^{2}-15/4k^{4}+64/5\pi k^{5}-5/4k^{6}}{1440}}^{}\right]}^{1/2}\\ L_{f}=\frac{V_{0}}{I_{0}f_{\text{sw}}}{\left\{K\frac{V_{\text{dclink}}}{V_{0,\text{av}}}\left[1+4\pi^{2}{\left(\frac{f}{f_{\text{sw}}}\right)}^{2}K\frac{V_{\text{dclink}}}{V_{0,\text{av}}}\right]\right\}}^{1/2}\\ C_{f}=\frac{V_{\text{dclink}}}{L_{f}{f_{\text{sw}}^{2}}^{}V_{0,\text{av}}}, \end{array}$$

where *k* (modulation index) = 1, *V*_0_ (Load Voltage) = 220 V,  *I*_0_ (Load Current) = 4.58 A,  *f* (fundamental frequency) = 50 HZ,  *f*_sw_ (switching frequency) = 2 kHZ,  *V*_0,av_ (total harmonic load voltage) = 5% of *V*_0_,  *L*_*f*_ is the inductance of filter,  and *C*_*f*_ is the capacitance of filter.

### C.

See Tables 1, 2, 3, and 4.

### D.

Using the literature [23, 25, 27, 29, 30], expression for the power due to the air pressure term can be obtained. This power term is derived in [23, 25] but present here for completeness. According to the above literature, it is clear that the power available at the wave turbine consists of two terms: air velocity term *P*_*a*_ and air pressure term *P*_pt_. The power *P*_pt_ mainly depends on the volume of air flow rate *Q* across the turbine and the pressure gradient. So power due to the air pressure term can be define as [23]

(D.1) $$\begin{array}{c} P_{\text{pt}}=\left(p_{2}-p_{0}\right)\times Q, \end{array}$$

where, from the continuity equation,

(D.2) $$\begin{array}{c} Q=A_{1}\times V_{1}=V_{2}\times A_{2}. \end{array}$$

The exhausted pressure *p*_0_ is assumed to be constant and ambient, even close to the turbine. The upstream inner pressure *p*_2_ near the turbine is related to the pressure within the air chamber by the Bernoulli energy equation:

(D.3) $$\begin{array}{c} p_{2}=p_{1}+0.5\times \rho \times \left(V_{1}^{2}-V_{2}^{2}\right)+\rho \frac{\delta }{\delta t}\left(\varphi_{1}-\varphi_{2}\right), \end{array}$$

where the velocity potentials *φ*_1_ and *φ*_2_ are approximated by

(D.4) $$\begin{array}{c} \varphi_{1}\approx V_{1}\eta_{1},\\ \varphi_{2}\approx V_{2}\eta_{2}. \end{array}$$

Based on the linear momentum equation theory, the pressure difference in (D.1) can be derived as

(D.5) $$\begin{array}{c} p_{2}-p_{0}\approx \rho \frac{A_{1}}{A_{2}}\frac{\delta \varphi_{1}}{\delta t}+\rho \frac{Q}{A_{2}}\left(V_{2}-V_{1}\right). \end{array}$$

From (D.1) and (D.5),

(D.6) $$\begin{array}{c} P_{\text{pt}}=\left[\rho \frac{A_{1}}{A_{2}}\frac{\delta \varphi_{1}}{\delta t}+\rho \frac{Q}{A_{2}}\left(V_{2}-V_{1}\right)\right]\times Q. \end{array}$$

But the rate of change of velocity potentials *φ*_1_ is

(D.7) $$\begin{array}{c} \frac{\delta \varphi_{1}}{\delta t}=-\frac{H_{\text{in}}^{2}}{\theta^{2}}\omega^{2}\left\{2\cos{\left(\omega t\right)}^{2}-1\right\}\times \text{si}{\text{n}}^{2}\left(\frac{\theta }{2}\right). \end{array}$$

Hence, power due to the air pressure term is

(D.8) Ppt=[−A1A2Hin2θ2ω2{2cos⁡⁡(ωt)2−1} × sin2(θ2)+QA2(V2−V1)]×Q×ρ.
